# Occupational exposure to dairy cattle influences virulence gene profiles of methicillin-resistant <em>Staphylococcus aureus</em> in humans: Antimicrobial resistance, enterotoxin, biofilm, and immune evasion determinants in Jordan

**DOI:** 10.14202/vetworld.2026.3246-3255

**Published:** 2026-07-28

**Authors:** Ibrahim M. Alzuheir, Mohammad H. Gharaibeh, Myassar O. Alekish, Ismail M. Malkawi, Farah R. Al Oudsi

**Affiliations:** 1 Department of Basic Veterinary Medical Sciences, Faculty of Veterinary Medicine, Jordan University of Science and Technology, Irbid, Jordan.; 2 Department of Veterinary Clinical Sciences, Faculty of Veterinary Medicine, Jordan University of Science and Technology, Irbid, Jordan.; 3 Department of Nutrition and Food Technology, Jordan University of Science and Technology, Irbid, Jordan.

**Keywords:** antimicrobial resistance, biofilm genes, dairy cattle, enterotoxins, Jordan, methicillin-resistant Staphylococcus aureus, occupational exposure, virulence gene profiles

## Abstract

**Background and Aim:**

*Staphylococcus aureus*, particularly methicillin-resistant *S. aureus* (MRSA), is a major zoonotic pathogen. This study assessed the prevalence of *S. aureus* and MRSA among individuals in close occupational contact with dairy cattle in Jordan and characterized the recovered MRSA isolates for antimicrobial resistance (AMR), enterotoxin, biofilm, and immune evasion gene profiles.

**Materials and Methods:**

A cross-sectional study was conducted using convenience sampling. Nasal swabs were collected from 100 cattle-exposed individuals and 100 non-exposed controls. *S. aureus* was identified by polymerase chain reaction (PCR) targeting the *nuc* gene, and MRSA was confirmed by *mecA* detection. Multiplex PCR assays screened for AMR genes, virulence gene profiles (VGP1–VGP8), biofilm genes, and immune evasion genes. Statistical comparisons between groups were performed using the chi-square test (p < 0.05).

**Results:**

*S. aureus* was isolated from 82/200 (41.0%) samples, with MRSA comprising 63/82 (76.8%) isolates (32 from exposed and 31 from non-exposed individuals). The *blaZ* gene was detected in 100% of exposed and 83.9% of non-exposed MRSA isolates. Tetracycline resistance genes (*tetK* and *tetM*) were highly prevalent in both groups. Biofilm-associated genes (*icaA* and *icaD*) were present in 100% of isolates. Significant differences (p < 0.05) were found for several virulence genes: *selr*, *hlg*, and *etd* were more common in exposed isolates, while *selp* and *sak* were more prevalent in non-exposed isolates. The *etd* gene was detected exclusively in exposed isolates (50%).

**Conclusion:**

Human MRSA isolates from both groups showed high AMR, biofilm formation, and virulence potential, indicating substantial zoonotic risk. Occupational exposure to dairy cattle was associated with distinct virulence gene signatures. These findings emphasize the need for continuous molecular surveillance and One Health interventions to mitigate MRSA transmission at the livestock–human interface in Jordan.

## INTRODUCTION

*Staphylococcus aureus* is an opportunistic ubiquitous bacterium colonized on human skin and mucosal surfaces, the environment, as well as in food-processing settings [1]. The high genetic diversity, multidrug resistance, biofilm formation, immune evasion, and persistent infections enhance its pathogenic potential and increase the difficulties of control [2, 3]. The production of heat-stable staphylococcal enterotoxins is a major cause of food poisoning, which remains active even after thermal processing of contaminated foods such as milk [4].

The emergence of methicillin-resistant *S. aureus* (MRSA) is alarming. It is traditionally classified into healthcare-associated MRSA (HA-MRSA), community-associated MRSA (CA-MRSA), and livestock-associated MRSA (LA-MRSA), each arising from distinct clonal lineages [5]. HA-MRSA circulates mainly in healthcare settings, CA-MRSA occurs in individuals with little or no healthcare exposure, and LA-MRSA is primarily linked to agricultural environments [6]. Some *S. aureus* strains produce biofilms mediated by the *ica* gene cluster, enhancing bacterial persistence and antimicrobial resistance (AMR) by limiting antibiotic penetration and facilitating genetic exchange [7]. Resistance to β-lactam antibiotics is commonly associated with the *blaZ* and *mecA* genes, which encode penicillinase and altered penicillin-binding proteins, respectively [2, 3].

*S. aureus* also carries a variety of virulence genes that contribute to disease severity, host invasion, immune evasion, and toxin production. Pathogenic MRSA strains encode a set of virulence gene profiles (VGP) that enhance their pathogenic potential, epidemiology, and clinical impact [8]. In addition, *S. aureus* encodes multiple enterotoxin genes, including *sea*–*see*, which are responsible for potent staphylococcal food poisoning [9]. These virulence factors mark *S. aureus* as a leading cause of foodborne illness globally [10]. Direct contact with livestock or cross-contamination during food handling and processing, particularly under poor hygienic conditions, are the main transmission routes of HA-MRSA, CA-MRSA, and LA-MRSA [11].

Despite the global recognition of MRSA as a significant zoonotic pathogen, there is limited information on how occupational exposure to dairy cattle influences MRSA carriage and molecular characteristics in humans, particularly in Middle Eastern countries such as Jordan. Most existing studies focus on MRSA in livestock or hospital settings, with few addressing the human–animal interface in dairy production systems. Data on the specific VGP, including enterotoxins, biofilm-associated genes, and immune evasion determinants, in individuals with direct dairy cattle contact versus non-exposed populations remain scarce. Furthermore, the potential bidirectional transmission and adaptation of MRSA strains at the livestock–human interface in Jordan, where dairy cattle production is a major agricultural sector, has not been systematically investigated. This knowledge gap hinders the development of targeted One Health interventions to mitigate zoonotic risks and AMR spread in pastoral and semi-intensive farming communities.

Therefore, this is the first report to investigate the prevalence of *S. aureus* and MRSA in human nasal samples, characterize their AMR and virulence-associated gene profiles, and assess the impact of dairy cattle exposure on the distribution of these determinants in Jordan. We hypothesized that occupational exposure to dairy cattle influences MRSA carriage and is associated with differences in AMR, virulence, enterotoxin, biofilm-associated, and immune evasion gene profiles compared with individuals without livestock exposure. The findings contribute to a better understanding of the epidemiology, zoonotic potential, food safety implications, and public health risks associated with MRSA within a One Health framework.

## MATERIALS AND METHODS

### Ethical approval

The study was reviewed and approved by the Institutional Animal Care and Use Committee (JUST–ACUC) and the Institutional Review Board (IRB) of Jordan University of Science and Technology (approval number 16/4/12/782, dated 12 December 2022). The protocol fully complied with the ethical principles of the Declaration of Helsinki (2013 revision), the Belmont Report, and the International Ethical Guidelines for Health-related Research Involving Humans (CIOMS, 2016). All participants provided written informed consent after receiving a detailed participant information sheet that explained the study purpose, procedures, potential risks (including minor discomfort from nasal swabbing), benefits, voluntary nature of participation, right to withdraw at any time without consequence, and measures for data confidentiality and anonymity.

Nasal swab collection was performed by trained medical or laboratory personnel using a standardized, non-invasive protocol under strict biosafety conditions. Participant data were de-identified using unique study codes, stored in password-protected databases accessible only to the principal investigator and authorized study team members, and handled in accordance with Jordanian data protection regulations and international standards (e.g., GDPR principles where applicable). No personally identifiable information was included in the manuscript or supplementary materials. The study posed minimal risk to participants, and appropriate provisions were made for referral to medical care if any adverse events occurred during or after sampling. Approval was obtained prior to the commencement of participant recruitment, and the study was conducted in full compliance with all local, national, and institutional ethical and legal requirements.

### Study period and location

A total of 200 human nasal swab samples were collected from dairy cattle farm environments and surrounding communities in northern Jordan (Irbid and Ramtha districts) between February and May 2023. These districts contain a high density of dairy cattle farms in northern Jordan, providing access to individuals with regular occupational exposure to dairy cattle. In addition, non-exposed participants were recruited from the same geographical area to minimize potential regional and environmental differences between the study groups.

### Study design

This cross-sectional study employed convenience sampling. Participants were categorized into two groups based on occupational exposure to dairy cattle and classified as cattle-exposed (occupational contact with dairy cattle) and non-exposed (no livestock contact). Both groups were recruited from the same area. The cattle-exposed group (n = 100) included individuals with regular daily direct contact with dairy cattle, such as farm workers, milkers, veterinarians, feed handlers, and farm owners. These participants were involved in activities including milking, animal handling, veterinary procedures, and contact with raw milk or farm equipment. The non-exposed group (n = 100) consisted of individuals from the same geographical area with no occupational or regular contact with livestock, including university students, administrative staff, and community residents.

Nasal swabs were collected from both nostrils by trained personnel using sterile cotton swabs moistened with sterile saline, following a standardized protocol to ensure consistency across all participants. The swabs were immediately placed into sterile transport tubes containing nutrient broth, properly labeled, and transported in insulated containers at 4°C to the Microbiology Laboratory, Department of Basic Veterinary Medical Sciences, Faculty of Veterinary Medicine, Jordan University of Science and Technology (JUST), within 2–3 hours of collection.

### Bacterial isolation and identification

Nasal swabs were processed as previously described by Gharaibeh *et al.* [12]. Standard selective and differential media (such as Mannitol Salt Agar and blood agar) were used, followed by phenotypic identification of *S. aureus* based on colony morphology, Gram staining, catalase and coagulase tests. Presumptive *S. aureus* isolates were identified using standard bacteriological methods, and MRSA was confirmed by PCR detection of the *nuc* and *mecA* genes.

### PCR detection of virulence and enterotoxin genes

All confirmed *S. aureus* isolates were screened for virulence-associated genes, enterotoxin genes, antimicrobial resistance (AMR) genes, and biofilm-related genes using ten multiplex PCR assays targeting biofilm genes, AMR determinants, hemolysins, and VGP1–VGP8, using primers and PCR amplification conditions as previously described [13–20].

### Statistical analysis

Data were entered and managed using Microsoft Excel and analyzed with GraphPad Prism version 10 (GraphPad Software, San Diego, CA, USA). Descriptive statistics were used to summarize the percentage of *S. aureus*, MRSA, and virulence- and AMR–associated genes, and results were expressed as frequencies and percentages. Comparisons between cattle-exposed and non-exposed groups were performed using the chi-square (χ²) test. A p-value of < 0.05 was considered statistically significant.

## RESULTS AND DISCUSSION

### MRSA isolation and confirmation

Among the 200 human nasal swab samples analyzed, *S. aureus* was detected in 42% (42/100) of cattle-exposed individuals and 40% (40/100) of non-exposed individuals. MRSA was confirmed by PCR detection of the species-specific *nuc* gene and the methicillin resistance gene *mecA*. Of the 82 *S. aureus* isolates recovered, 63 (76.8%) were identified as MRSA, including 32 isolates from cattle-exposed individuals and 31 from non-exposed individuals (Table 1).

**Table 1:** Prevalence of Staphylococcus aureus and methicillin-resistant S. aureus (MRSA) isolates from nostril samples of humans exposed and unexposed to dairy cattle.

**Sample source**	**Total samples (n)**	***S. aureus*** ** positive, n (%)**	**MRSA among ** ***S. aureus*** **, n (%)**
Humans (exposed)	100	42 (42.0)	32 (76.2)
Humans (non-exposed)	100	40 (40.0)	31 (77.5)
Total	200	82 (41.0)	63 (76.8)

The high prevalence of MRSA in both exposed and non-exposed groups suggests that methicillin resistance is not restricted to farm environments. The comparable MRSA rates observed between the two groups indicate a substantial contribution of CA-MRSA circulation, which has been increasingly reported in Jordan and neighboring countries [21, 22]. These findings support growing evidence that MRSA is now widely established in community settings beyond healthcare and agricultural environments [22, 23].

The detection of MRSA among cattle-exposed individuals supports the potential role of contact with livestock and their products in the distribution of MRSA strains. Close human–animal contact enhances dissemination through direct contact or via indirectly contaminated farm equipment and inadequate hygienic practices [23]. The adaptability of MRSA to multiple hosts, partly mediated by mobile genetic elements such as plasmids, further enhances its transmission potential [24]. Previous molecular studies have shown that certain *S. aureus* lineages, including ST8, circulate between humans and livestock, highlighting the potential bidirectional transmission [8]. The direct transmission of LA-MRSA to humans is less frequent than CA-MRSA; however, livestock-exposed workers may serve as a key reservoir or source for MRSA dissemination. The genetic relatedness between LA-MRSA and CA-MRSA strains further suggests the zoonotic potential of LA-MRSA [25, 26]. These findings confirm the necessity for continuous surveillance and characterization of MRSA in human populations with various levels of livestock exposure, including farmers, milkers, veterinarians, and individuals without direct livestock contact. Understanding the relative contributions of community and occupational transmission is critical for designing targeted prevention strategies and implementing integrated control measures under a One Health framework [26, 27].

### Detection of AMR genes

**Figure 1:**
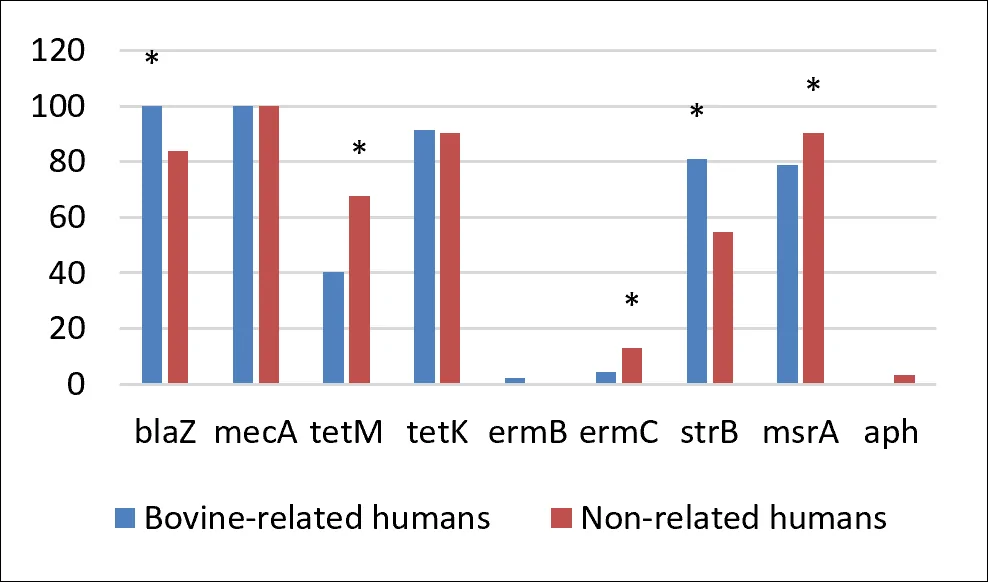
Distribution of antimicrobial resistance genes among methicillin-resistant *Staphylococcus aureus* isolates from human (cattle-exposed) and human (non-exposed) groups. Bars represent the percentage of isolates harboring each resistance gene. Statistical significance was determined using χ² or Fisher’s exact test; p < 0.05.

All confirmed MRSA isolates from human nasal swabs were screened for key AMR genes, including *blaZ*, *mecA*, *tetK*, *tetM*, *ermB*, *ermC*, *strB*, *msrA*, and *aph*. The percentage and distribution of these genes differ among cattle-exposed and non-exposed individuals, reflecting variations in exposure and selective pressures across the groups (Figure 1).

The β-lactamase gene *blaZ* was the most prevalent AMR determinant detected, occurring in 100% of isolates from cattle-exposed humans and 83.9% of isolates from non-exposed individuals, with a significant difference between groups (p < 0.05). The ubiquitous presence of *blaZ* confirms the widespread resistance of *S. aureus* to penicillin and reflects the long-standing and often inappropriate use of β-lactam antibiotics in both human and veterinary medicine [28]. Tetracycline resistance genes (*tetM* and *tetK*) were frequently detected in isolates from both groups. Among cattle-exposed human isolates, *tetM* and *tetK* were identified in 40.4% and 91.5% of isolates, respectively, while corresponding frequencies among non-exposed human isolates were 67.7% (*tetM*) and 90.3% (*tetK*), with no statistically significant differences between groups. The high percentage of tetracycline resistance genes in both populations suggests shared or overlapping exposure pathways. This likely reflects the extensive use of tetracyclines for prophylactic and therapeutic purposes in livestock production, as well as their continued use in human medicine [29].

Macrolide resistance genes (*ermB* and *ermC*) were comparatively infrequent. *ermB* was detected in 2.1% of cattle-exposed human isolates and was absent in non-exposed human isolates. *ermC* was found in 4.3% and 12.9% of isolates, respectively, with a significant difference between groups (p < 0.05). In contrast, *strB* (conferring streptomycin resistance) and *msrA* (encoding a macrolide efflux pump) were highly prevalent, with 80.9% and 78.7% in cattle-exposed isolates and 54.8% and 90.3% in non-exposed isolates, with a significant difference between groups (p < 0.05). The widespread occurrence of *strB* and *msrA* suggests potential cross-resistance between aminoglycosides and macrolides, which may complicate treatment strategies in both human and veterinary settings [29, 30]. The aminoglycoside-modifying enzyme gene *aph* was not detected in cattle-exposed human isolates but was present in 3.2% of non-exposed human isolates, suggesting the potential for horizontal gene transfer and genetic exchange between strains from different sources [31].

Overall, the observed distribution of AMR genes of MRSA isolates indicates a high percentage of β-lactamase, tetracycline, streptomycin, and macrolide resistance among MRSA isolates from both groups. Similar AMR gene patterns in exposed and non-exposed humans suggest that MRSA resistance is not only linked to direct cattle contact. Instead, resistant strains likely circulate through multiple routes, including food chains, environment, healthcare settings, and human-to-human transmission [29]. This indicates widespread dissemination of AMR determinants beyond farms into the community. It reflects the interconnected role of humans, animals, and the environment in AMR spread. Therefore, a One Health antimicrobial stewardship approach is urgently needed in Jordan.

The pattern of AMR gene detection between cattle-exposed and non-exposed humans shows the interaction between livestock, humans, and the environment in shaping the AMR profiles of MRSA strains and raises the urgent need for an integrated AMR stewardship program under a One Health approach for MRSA control in Jordan.

### Detection of virulence, biofilm, and enterotoxin genes

A total of 63 (32 cattle-exposed and 31 non-exposed) MRSA isolates were screened for virulence-associated genes (VGP1–VGP8), hemolysins, immune evasion cluster genes, and biofilm-associated genes (Table 2).

**Table 2:** Comparative distribution of virulence gene profiles (VGP1–VGP8), hemolysins, immune evasion, and biofilm-associated genes in dairy cattle exposed and non-exposed human methicillin-resistant Staphylococcus aureus isolates in Jordan.

**Virulence gene profile (VGP)**	**Gene**	**Exposed % (95% CI)**	**Non-exposed % (95% CI)**	**χ²**	**p-value**
VGP1	*sea*	94 (79–99)	89 (72–97)	0.5	0.5
	*seb*	0 (0–11)	0 (0–11)	—	—
	*sec*	3 (0–16)	0 (0–11)	1	0.31
	*sed*	28 (15–46)	35 (20–53)	0.4	0.54
	*see*	0 (0–11)	0 (0–11)	—	—
VGP2	*seg*	97 (84–100)	96 (80–100)	0.1	0.82
	*seh*	56 (39–72)	55 (37–72)	0	0.92
	*sei*	100 (89–100)	100 (89–100)	—	—
	*sej*	94 (79–99)	90 (73–98)	0.4	0.53
	*selp*	59 (41–75)	97 (83–100)	12	<0.001
VGP3	*selk*	0 (0–11)	0 (0–11)	—	—
	*selm*	91 (75–98)	94 (79–99)	0.2	0.65
	*selo*	97 (84–100)	100 (89–100)	1	0.31
	*tst*	75 (57–88)	84 (66–94)	0.8	0.39
VGP4	*sell*	6 (1–19)	6 (1–19)	0	1
	*selq*	0 (0–11)	0 (0–11)	—	—
	*selr*	97 (84–100)	45 (28–63)	20	<0.001
VGP5	*seln*	97 (84–100)	94 (79–99)	0.3	0.6
	*selu*	91 (75–98)	97 (83–100)	1.2	0.27
VGP6 (Hemolysins)	*hla*	91 (75–98)	97 (83–100)	1.2	0.27
	*hlb*	31 (17–49)	55 (37–72)	3.8	0.05
	*hld*	97 (84–100)	97 (83–100)	0	1
	*hlg*	100 (89–100)	71 (52–86)	11	0.001
	*hlg2*	66 (47–81)	45 (28–63)	2.8	0.09
VGP7	*eta*	0 (0–11)	0 (0–11)	—	—
	*etb*	0 (0–11)	0 (0–11)	—	—
	*etd*	50 (33–67)	0 (0–11)	22	<0.001
VGP8	*lukM*	0 (0–11)	0 (0–11)	—	—
	*lukED*	97 (84–100)	94 (79–99)	0.3	0.6
Immune evasion cluster	*chp*	100 (89–100)	94 (79–99)	1.9	0.16
	*sak*	81 (64–92)	97 (83–100)	4.2	0.04
	*scn*	91 (75–98)	94 (79–99)	0.2	0.65
Biofilm	*icaA*	100 (89–100)	100 (89–100)	—	—
	*icaD*	100 (89–100)	100 (89–100)	—	—

Among enterotoxin genes grouped within VGP1, the *sea* gene was highly prevalent in both exposed (94%) and non-exposed isolates (89%), with no significant difference (p = 0.5). Other genes, including *sec* and *sed*, showed moderate to low percentages without statistical significance (p > 0.05), while *seb* and *see* were absent in both groups. The percentages of these genes did not significantly vary among cattle-exposed and non-exposed isolates, reflecting diverse virulence profiles and suggesting no potential effect of dairy cattle exposure on adaptation and transmission dynamics.

In VGP2, the majority of genes (*seg*, *seh*, *sei*, and *sej*) were widely distributed in both groups with no significant differences (p > 0.05). However, *selp* was significantly more prevalent in non-exposed isolates (97%) compared to exposed isolates (59%) (χ² = 12.1, p < 0.001). Within VGP3, genes (*selm*, *selo*, and *tst*) were common in both groups with no statistically significant differences. The VGP3 genes are key mediators of staphylococcal food poisoning; the high percentage of these virulence genes among both cattle-exposed and non-exposed isolates suggests that they are well established within human-associated MRSA populations, indicating that their distribution may persist independently of livestock exposure [32]. This conserved percentage of VGP 1, 2, and VGP3-associated enterotoxins among circulating human strains suggests that these virulence factors are likely maintained through community transmission, rather than being influenced by occupational exposure to dairy cattle. These findings contest with previous reports of the stability of enterotoxin gene clusters among human-adapted *S. aureus* lineages. Previous reports on genetic analyses also showed that enterotoxin gene clusters and individual enterotoxin genes (e.g., *sea*, *seg*, *sei*) are widely distributed across diverse *S. aureus* lineages and are conserved in human-associated strains, suggesting they are maintained through community transmission rather than solely by specific host exposures [33].

In contrast, VGP4 revealed a marked difference for *selr*, which was significantly more prevalent in exposed isolates (97%) than in non-exposed isolates (45%) (χ² = 19.5, p < 0.001). This finding suggests that occupational contact with dairy cattle may influence the acquisition or persistence of specific enterotoxin gene variants. Enterotoxin-like genes such as *selr* are frequently located on mobile genetic elements, including pathogenicity islands, which facilitates their dissemination among MRSA populations circulating at the human–animal interface [34]. Previous molecular studies have reported the presence of *selr* in *S. aureus* isolates from dairy cattle, raw milk, and dairy products, indicating that this gene may be enriched in livestock-associated or food-related strains [34]. The high occurrence of *selr* among cattle-exposed individuals supports the hypothesis that repeated contact with colonized animals or contaminated farm environments may promote zoonotic spillover or genetic exchange between livestock-associated and human-adapted *S. aureus* lineages [35]. Therefore, the significantly higher detection of *selr* in cattle-exposed isolates underscores its potential relevance as a marker of occupational exposure and highlights the importance of monitoring non-classical enterotoxin genes in One Health–based surveillance programs. Other VGP4 genes showed no variation between groups.

For VGP5, both *seln* and *selu* genes, part of the enterotoxin-like (SEl) family, were highly prevalent in the two groups without significant differences (p > 0.05), indicating these genes circulate in the community as well as in livestock settings [36]. This finding may enhance colonization, immune evasion, and zoonotic transmission, highlighting the need for continuous surveillance.

The VGP6 group encodes hemolysin cytotoxic proteins that lyse erythrocytes and immune cells, facilitating bacterial survival, tissue invasion, and pathogenesis [20, 37]. The results showed that these genes were broadly distributed among isolates. The *hla* and *hld* genes showed high percentages (>90%) in both groups with no significant differences. However, *hlg* was significantly more common in exposed isolates (100%) compared to non-exposed isolates (71%) (χ² = 10.6, p = 0.001). The *hlb* gene was more frequent in non-exposed isolates (55%) than in exposed isolates (31%) (χ² = 3.8, p = 0.05). No significant difference was observed for *hlg2* (p = 0.09). The overall higher percentage of VGP6 in cattle-exposed isolates (p < 0.05) indicates that occupational exposure to livestock may contribute to colonization by more virulent MRSA strains, consistent with findings in other human and animal studies [38].

VGP7 comprises exfoliative toxins (*eta*, *etb*, and *etd*), which are proteases that cause loss of keratinocyte cohesion in the superficial epidermis and sometimes mucous membranes. Exfoliative toxins *eta* and *etb* mainly cause staphylococcal scalded skin syndrome (SSSS), while *etd* is linked to other skin infections [37]. Our results showed that the *etd* gene was detected in 50% of exposed isolates but was completely absent in non-exposed isolates, representing a highly significant association (χ² = 22.4, p < 0.001). In contrast, *eta* and *etb* were not detected in either group. The absence of *eta* and *etb* in both human groups suggests that SSSS-associated toxins are not circulating in these populations, which is expected since SSSS is mainly a pediatric disease and rarely linked to adults exposed to livestock [37, 38]. The detection of *etd* exclusively in individuals exposed to cattle indicates a potential zoonotic transmission of *etd*-producing MRSA from cattle to humans. The finding also highlights that occupational exposure to livestock can influence the carriage of specific virulence factors, suggesting that humans in close contact with cattle may serve as reservoirs or temporary carriers of *etd*-positive strains [38].

VGP8 included leukocidin genes. The *lukED* gene was highly prevalent in both exposed (97%) and non-exposed (94%) isolates with no significant difference (p = 0.6), whereas *lukM* was absent in both groups. The *lukM* gene, typically associated with ruminant-specific strains, was not detected in human samples, suggesting a host-adapted role in bovine infections [39].

Biofilm-associated genes (*icaA* and *icaD*) were detected in 100% of isolates across both cattle-exposed and non-exposed isolates, indicating universal biofilm-forming potential with no observable variation.

Among the immune evasion cluster genes, *chp* and *scn* encode the chemotaxis inhibitory protein of *S. aureus* (CHIPS) and the staphylokinase (SAK) enzyme and were highly prevalent (>90%) in both groups without significant differences. However, *sak* was significantly more common in non-exposed isolates (97%) compared to exposed isolates (81%) (χ² = 4.2, p = 0.04). None of the isolates carried the *scn* gene encoding the staphylococcal complement inhibitor protein (SCIN).

Overall, our findings show a broad distribution of virulence-, biofilm-, and immune evasion–associated genes among human MRSA isolates. Statistically significant differences between cattle-exposed and non-exposed groups were observed for selected enterotoxin gene profiles, hemolysin-associated gene clusters, the leukocidin gene *lukM*, and capsular polysaccharide genes (*cap5* and *cap8*). These differences suggest that occupational exposure to cattle may be associated with variations in the virulent gene repertoire of human MRSA isolates. The detection of multiple virulence and enterotoxin genes among MRSA isolates indicates their potential to cause a wide range of clinical outcomes, from mild skin and soft tissue infections to more severe invasive diseases. The concurrent presence of virulence determinants and AMR genes may contribute to increased bacterial fitness and persistence, thereby complicating infection management in both human and animal hosts. Furthermore, the occurrence of shared virulence-associated genes among cattle-exposed and non-exposed human isolates supports the importance of reinforcing hygiene measures during milking, milk processing, and food handling. In addition, continuous molecular surveillance is warranted to better characterize transmission dynamics and to monitor the emergence of virulent MRSA lineages within a One Health framework.

## CONCLUSION

This study provides the first evidence from Jordan on the influence of occupational exposure to dairy cattle on MRSA in humans. Key results showed a high overall prevalence of *S. aureus* (41.0%) and MRSA (76.8% of *S. aureus* isolates), with comparable rates between cattle-exposed and non-exposed groups. MRSA isolates exhibited high levels of AMR genes (e.g., *blaZ* in 100% of exposed isolates) and universal biofilm-forming capacity (*icaA* and *icaD* in 100% of isolates). VGPs were broadly similar, but significant differences were observed: *selr*, *hlg*, and *etd* were more prevalent in cattle-exposed individuals, while *selp* and *sak* were more common in non-exposed individuals.

These findings highlight the zoonotic potential of MRSA at the human–livestock interface in Jordan’s dairy sector and the need for enhanced hygiene practices during milking, animal handling, and milk processing. The high AMR and virulence gene burden in both groups underscores the importance of integrated antimicrobial stewardship programs under a One Health framework to reduce transmission risks to humans, animals, and the environment.

Strengths of the study include the comparative design with well-defined exposed and non-exposed groups from the same geographical area, comprehensive molecular characterization of multiple gene categories (AMR, enterotoxins, biofilm, immune evasion), and the use of standardized PCR methods. This is the first such investigation in the Middle East focusing on dairy cattle occupational exposure.

The study was limited by convenience sampling, which may affect generalizability, and the cross-sectional design, which does not establish causality. Molecular characterization did not include whole-genome sequencing or clonal typing, and the sample was restricted to nasal carriage rather than clinical infections.

Future studies should incorporate larger, longitudinal sampling across different regions of Jordan, whole-genome sequencing to track transmission and adaptation, and inclusion of environmental and livestock samples within a full One Health approach. Expanding surveillance to other livestock species and assessing clinical outcomes would further strengthen understanding of MRSA epidemiology.

In conclusion, occupational exposure to dairy cattle is associated with distinct virulence gene signatures in human MRSA isolates, while high AMR and biofilm capacity are widespread. Continuous molecular surveillance and strengthened One Health interventions are essential to mitigate zoonotic risks and preserve the effectiveness of antimicrobials in Jordan and similar settings.

## DATA AVAILABILITY

The data generated during the study are included in the manuscript.

## GENERATIVE AI DECLARATION

The authors declare that no generative artificial intelligence (AI) or AI-assisted technologies were used in the writing, analysis, or preparation of this manuscript.

## AUTHORS’ CONTRIBUTIONS

IMA: Investigation, interpretation of data, writing, reviewing, and editing of the manuscript. MHG: Formal analysis, editing and reviewing the original draft. MOA: Conceptualization, supervision, project administration, and reviewing of the manuscript. IMM: Methodology and review and editing. FRAO: Methodology, formal analysis, and data curation. All authors have read and approved the final version of the manuscript.
